# A Local Committee Urged to Continue

**Published:** 1923-05

**Authors:** 


### A LOCAL COMMITTEE URGED TO CONTINUE.

The Lancashire Voluntary Hospitals Committee, which held four Quarter Sessional Conferences in the area, has issued an interesting report. The Conference found that there was no uniformity in the assistance received from local authorities, and some hospitals feared that rate-aid might lead to rate-control, but all agreed that Municipal Corporations might be asked, in their capacity of large employers of labour, to make annual contributions to the hospitals in their districts in return for the hospital service rendered to their employees, as many large firms are in the habit of doing. It was decided to ask the Ministry of Health to make this suggestion to the Corporations. Many Lancashire hospitals were found to be asking non-contributing patients to pay from 6s. a day downwards, and an hospital of seventy beds has received £2,000 in one year from this source.

One-third of these patients paid the 6s. ; one-third paid something less down to 1s. a day, and the remaining third nothing. The principle of weekly contribution schemes was unanimously approved, and the work-people's representatives were urged to help to extend the plan by explaining it directly to other wage-earners. It was considered advisable to try to organise support from rural areas on some more organised lines than that of individual collectors and personal calls. One-tenth of the approved societies or registered branches in the country contribute to the maintenance of insured persons when in hospital, and it was thought that this source of income might become very productive if hospital committees who receive insured persons would make a practice of ascertaining to which societies the patients belong. While the Conferences thought co-operative purchase was not practicable, because hospitals must spend much of their money in the places from which they receive it, it was thought that the Lancashire Voluntary Hospitals Committee would do useful work if it obtained schedules of the prices paid by the different hospitals in the area for the different classes of goods and supplies, and distribute the information to the secretaries. This is being done.

It was suggested that well-equipped hospitals could help others by giving such facilities as were possible for the use of their apparatus, and that those with vacant beds should accept patients from others with a long waiting list. The loan of a skilled radiologist from a larger institution was proposed for the benefit of small hospitals which have X-ray installations. The conferences were so successful in bringing the hospitals together that it was hoped that the Lancashire Committee would continue them. The argument against co-operative purchase is interesting, for only the other day we noticed that a certain Chamber of Commerce insisted that the local hospital should inquire into the prevalent belief that many of its purchases, which could be made locally, were made outside the town. The hospital was able to reassure the tradesmen, but the occurrence proves the difficulty of co-operative purchase, which even the action of a London body like the Red Cross would hardly solve.

